# Recent Research on Biosensors for Biomarker Detection Related to Neurological Disorders Utilizing Surface Engineering Strategies

**DOI:** 10.3390/bios16100541

**Published:** 2026-09-28

**Authors:** Yejin Lee, Minji Seo, Sengyeon Pyo, Yumi Kim, Hye Kyu Choi, Minkyu Shin, Jinho Yoon

**Affiliations:** 1 Department of Biomedical-Chemical Engineering, The Catholic University of Korea, Bucheon-si 14662, Gyeonggi-do, Republic of Korea; bioyjlee@catholic.ac.kr (Y.L.); engelminji@catholic.ac.kr (M.S.); seungyeon1464@catholic.ac.kr (S.P.); dbalrla0313@catholic.ac.kr (Y.K.); 2 Department of Biotechnology, The Catholic University of Korea, Bucheon-si 14662, Gyeonggi-do, Republic of Korea; 3 Department of Chemistry and Chemical Biology, Rutgers, The State University of New Jersey, Piscataway, NJ 08854, USA; hkc40@chem.rutgers.edu; 4 Department of Chemical Engineering, Research Center of Chemical Technology, Hankyong National University, Anseong-si 17579, Gyeonggi-do, Republic of Korea

**Keywords:** biosensor, neural biosensor, surface engineering, neurological disorders, neural biomarkers

## Abstract

Surface engineering strategies have been widely used in various technological fields. Particularly, they have been widely utilized in the biosensing field, to improve interfacial properties and facilitate molecular recognition. Among the various targets of biosensors, in recent years are neurological disorders, which have become a major health challenge globally, increasing the demand for accurate and early detection of neural biomarkers. Despite significant advances in biosensors for the detection of neural biomarkers, traditional biosensors often suffer from low sensitivity and selectivity for the precise detection of neural biomarkers. In this regard, surface engineering strategies hold huge potential and utility to address above issues of biosensors. Therefore, a comprehensive understanding of surface engineering strategies and their impact on neural biosensing is essential for the diagnosis of neurological disorders. In this review, we present recent advances in the development of biosensors for neural biomarker detection that incorporate surface engineering strategies. We first discuss surface engineering strategies applicable in biosensors and their underlying mechanisms. Next, recent surface-engineered biosensors for neural biomarker detection are discussed, configured according to the environmental conditions of biomarkers. We anticipate that this review will provide valuable insights into the development of novel biosensors for the early diagnosis of neurological disorders.

## 1. Introduction

Surface engineering strategies have been widely used in diverse technological fields, from battery electrodes and electrocatalysts to drug delivery systems [1,2,3]. To date, numerous surface engineering strategies have been developed and utilized based on the widely known gold–thiol interaction to EDC/NHS coupling [4,5]. These might appear to be somewhat dated, using an already long-established approach, yet such strategies have huge potential and are applicable in the biosensing field through the enhancement of interfacial properties and the facilitation of molecular recognition [6,7]. In developing biosensors, surface engineering strategies can be utilized for the efficient introduction of biosensor probes, the fabrication of functional electrodes, and the creation of polymer-based scaffolds capable of incorporating living biological materials such as cells and organoids to detect biomarkers from living organism mimicking systems [8,9]. The advantages of surface engineering strategies have been applied in developing biosensors in nanostructured interface engineering, molecular recognition engineering, conductive biointerface engineering, and antifouling and passivation engineering, to overcome limitations of conventional biosensors such as low sensitivity or selectivity, leading to enhanced target detection capability [10,11,12].

Recently, in the field of biosensors, tremendous interest in neurological disorders such as Alzheimer’s and Parkinson’s has been growing worldwide [13,14]. Since the global occurrence rates of these diseases have increased significantly, they are recognized as critical issues in the biomedical field [15,16]. However, for achieving effective medical treatment for neurological disorders, the accurate detection of biomarkers associated with neurological disorders is crucial [17]. Therefore, there is a growing interest in the accurate detection of neural biomarkers using biosensors such as neurotransmitters and miRNA for accurate diagnosis of neurological disorders [18,19]. Even though research on the development of neural biosensors, which are biosensors capable of detecting neural biomarkers, has been conducted extensively, traditional neural biosensors have had limitations in terms of sensitivity and reproducibility, falling short of the biosensing capabilities for the early diagnosis of neurological disorders. To enable reliable neural biomarker biosensing, biosensors require high sensitivity and selectivity while maintaining stability and functionality in complex biological environments. In this regard, surface engineering strategies can suggest a new direction for the development of accurate neural biosensors. Accordingly, various biosensors capable of detecting neural biomarkers with high sensitivity have recently been reported based on surface engineering strategies [20,21].

From this point of view, a comprehensive understanding of surface engineering strategies and their applications in the development of neural biosensors is essential for guiding future biosensor development for the clinical diagnosis of neurological disorders. In this review, we present recently reported studies about advances in biosensors for neural biomarker detection using surface engineering strategies (Figure 1). First, the most widely used surface engineering strategies applicable in the development of biosensors and their underlying mechanisms are discussed, such as nanostructured interface engineering, molecular recognition engineering, conductive biointerface engineering, and antifouling and passivation engineering. Subsequently, we focus on recent neural biosensors employing these strategies, organized according to the level of biological targets, from soluble neural biomarkers to cell-level systems and beyond-the-cell platforms based on spheroids and organoids. This review will provide valuable insights into the development of accurate surface-engineered neural biosensors to be applied for the diagnosis of neurological disorders in the early stage.

## 2. Surface Engineering Strategies for Biosensors

The performance of biosensors can be significantly improved through the stable immobilization of sensing probes, efficient electron transfer, and the suppression of nonspecific adsorption. These interfacial properties are widely recognized as critical factors determining the sensitivity and selectivity of biosensors [22,23,24]. To this end, a variety of surface engineering strategies have been utilized to optimize the interactions between biomolecules and electrode interfaces [25,26]. In this chapter, we introduce representative surface engineering strategies employed in biosensors and discuss their functional roles.

### 2.1. Nanostructured Interface Engineering

MXenes are two-dimensional (2D) multifunctional nanomaterials that have attracted attention as representative materials for electrode surface engineering due to their high electrical conductivity, abundant hydrophilic surface functional groups, and large specific surface area [27,28]. In particular, the high ion accessibility and rapid charge transport facilitate efficient electron transfer at the electrode–electrolyte interface, enabling highly sensitive electrochemical biosensing [29,30]. In one study, A gold nanoparticle (AuNP)-MXene nanocomposite was incorporated into a screen-printed electrode (SPE) [31]. The resulting interface exhibited rapid charge-transfer kinetics and high electrical conductivity, enabling sensitive dopamine detection. In addition to MXenes, the metal-organic framework (MOF), composed of metal nodes interconnected by organic linkers, has emerged as a candidate for use of electrode surface engineering materials due to its high specific surface area and tunable porous structures [32,33,34]. For example, an MOF-based neuron was developed in which the porous structure promoted dopamine diffusion toward the channel surface [35]. Oxidation of dopamine enhanced the faradaic current, which was in turn converted into a gating signal of the transistor. Depending on the dopamine concentration, the MOF surface itself functioned as a chemical switching input, tuning the channel threshold accordingly. This interface was further integrated with a microcontroller unit (MCU) to emulate the integrate-and-fire model, encoding the degree of dopamine-mediated gating directly into the spike number and width. Zeolitic imidazolate framework-8 (ZIF-8), another representative MOF, has also been utilized for encapsulating functional molecules based on the size compatibility between its pores and guest molecules. For instance, an ionophore with a molecular diameter (9.8 Å) between the cavities (11.6 Å) and pore dimensions (3.4 Å) of ZIF-8 allowed the ionophore to be physically confined while being further stabilized through Zn-N coordination [36]. This strategy provided long-term stability for ion biosensing in the brain. Similarly, Amine-modified ZIF-8 enabled the encapsulation of AuNPs through a locking in situ reduction series strategy, preventing excessive aggregation and particle growth [37]. In addition, Au@ZIF-8 exhibited an increased surface area compared with pure ZIF-8, offering more accessible active sites for dopamine adsorption and electrocatalytic activation. The developed sensor demonstrated its applicability to dopamine detection in fetal bovine serum. Covalent organic frameworks (COFs), which exhibit porous architectures similar to MOFs, have also been explored for surface engineering. For example, a COF synthesized from 2,4,6-trihydroxybenzene-1,3,5-tricarbaldehyde (Tp) and *p*-phenylenediamine (Pa) as monomers (TpPa COF) was grown on a carbon fiber microelectrode [38]. Its nanopores closely corresponded to the size of the dopamine oxidation intermediate, allowing the intermediate to be spatially confined and thereby inhibiting polymerization of dopamine on the electrode surface. This architecture enabled stable and real-time monitoring of dopamine in the brain of a Parkinson’s disease (PD) model mouse. Beyond MOF and COF, carbon-based nanomaterials have been widely engineered at the nano scale to enhance the surface area and conductivity of the sensing interface. Nanoporous graphene-based thin films were fabricated by stacking hydrothermally reduced graphene oxide flakes into a nanoporous network, achieving a high surface-to-volume ratio (Figure 2a) [39]. This structure resulted in an enhanced interfacial capacitance, over 10^4^-fold higher than that of non-porous single-layer graphene. In addition, the electrode also demonstrated robust mechanical stability under ultrasonic water-bath sonication (200 W and 300 W, 15 min each), with no delamination or cracking observed. When chronically implanted in the mouse prefrontal cortex for more than 90 days, it enabled reliable auditory evoked potential (AEP) recording with sustained signal quality. In addition, carbon nanotubes (CNTs) have been widely employed in electrode surface engineering because of their high carrier mobility, intrinsic electrical conductivity, and nanoscale architecture [40]. A surface-enhanced Raman scattering (SERS)-based sensor fabricated from CNT sheets decorated with AuNPs has been reported [41]. Oxygen plasma treatment loosened the CNT bundles by introducing oxygen-containing functional groups onto the walls, providing sufficient interstitial space for subsequent AuNP deposition. Dimethyl sulfoxide (DMSO) further promoted debundling via van der Waals interactions with these functional groups, while its strong S-Au interaction offered additional nucleation spots for Au clusters and suppressed their aggregation. The uniform decoration of AuNP effectively reduced variations in SERS intensity, leading to improved signal reproducibility. These nanostructured interfaces highlight the importance of the nanoscale control of pore structures and surface architectures in improving electron transfer, active-site accessibility, and mechanical stability.

### 2.2. Molecular Recognition Engineering

Antibody-based surface engineering represents one of the most widely used approaches to molecular recognition in biosensors, enabling the selective capture of target molecules through the high affinity and specificity of antibody–antigen interactions [42,43]. Accordingly, precise control over antibody orientation and immobilization on the biosensing surface is essential for enhanced sensing performance. Conventional immobilization methods such as physical adsorption can lead to random antibody orientation, while covalent bonding offers improved stability but remains sensitive to environmental conditions. Both approaches, however, ultimately reduce the probability of antibody–antigen binding [44,45]. To address this issue, a biosensor was developed that aligns polar antibodies (immunoglobulin G [IgG]) under an applied electric field [46]. Because the antigen-binding fragment (Fab) and the fragment crystallizable region (Fc) of IgG carry opposite charges, the antibody orientation can be controlled under an electric field. While immobilized IgG density remained similar, the aspect ratio increased from ~1.30 to ~1.59, enabling sensitive detection of antigen amyloid-beta (Aβ) 1–42 even in standard plasma. In addition to orientation, distance from the sensing surface is another critical parameter. Debye length (*λ_D_*) describes the distance over which the electrostatic effects of charged molecules persist before being screened by surrounding ions [47]. In physiological solutions such as blood, *λ_D_* is reduced to approximately 1 nm; however, most probe molecules such as whole IgG antibodies (~15 nm) position their antigen-binding events far beyond the *λ_D_* [48]. To address this, a graphene field-effect transistor (GFET) biosensing platform was developed utilizing F(ab’)_2_ fragments as probe molecules [49]. These fragments lack the Fc region and are generated through pepsin-mediated enzymatic cleavage of whole IgG antibodies. This size reduction decreased the effective layer thickness from 1.36 nm for IgG to 0.59 nm for F(ab’)_2_, positioning binding events closer to the graphene surface while also increasing receptor density per unit area. The platform was also validated by measuring neurofilament light (NfL) in plasma from traumatic brain injury (TBI) patients. Among various biomolecules applicable in molecular recognition engineering, the streptavidin–biotin interaction is one of the most widely used biomolecules that can provide a robust interface for the stable immobilization of biotinylated biomolecules through its ultrahigh binding affinity (Kd ≈ 10^−14^ M) and near-irreversible interaction [50,51,52]. For example, two spectrally distinct quantum dots were each conjugated with streptavidin and pre-assembled with different biotinylated secondary antibodies, generating two independent fluorescent probes for multiplexed detection [53]. The strong streptavidin–biotin interaction kept each probe intact throughout the assay, preventing dissociation or antibody exchange between probes. Because streptavidin is a tetrameric protein with four biotin-binding sites, each streptavidin-conjugated quantum dot can bind multiple biotinylated antibodies, contributing to signal amplification. The detection limit achieved with this approach was 6-fold lower than that of a conventional immunoassay without the streptavidin–biotin system. Also, an aptamer is another attractive biomolecule used in surface engineering strategy; it is a nucleic acid-based recognition molecule that undergoes conformational changes upon specific target binding, enabling efficient signal transduction [54,55]. Its high thermal stability and ease of synthesis has made it an attractive alternative to antibodies for molecular recognition engineering [56,57]. For instance, a nanopipette sensor was developed by modifying the nanopore (10 nm) with target-specific aptamers (Figure 2b) [58]. The negatively charged aptamers produced an asymmetric ion current. Upon target binding, the resulting conformational change altered the aptamer layer’s charge density and ion permeability, causing this current to shift. The sensor selectively distinguished dopamine from structurally similar molecules such as norepinephrine and dihydroxyphenylalanine. Due to the instability of aptamers under extreme pH and temperature conditions, the molecularly imprinted polymer (MIP) has attracted attention as a synthetic receptor generated by polymerizing monomers around a target molecule to create complementary binding cavities [59,60]. Recently, a dual electrochemical sensor based on MIP for the detection of nerve growth factor (NGF) and proNGF has been reported, exploiting the principle that the current signal decreases when the target rebinds to the cavity [61]. The MIP electrode exhibited imprinting factors of 30 (proNGF) and 26 (NGF), based on the current ratio between the MIP and non-imprinted polymer (NIP) electrodes, demonstrating the specific recognition capability. These findings emphasize that biosensor performance depends not only on the selection of recognition molecules but also on the controlled orientation, surface density, and stability of the immobilized recognition elements.

### 2.3. Conductive Biointerface Engineering

Poly(3,4-ethylenedioxythiophene) polystyrene sulfonate (PEDOT:PSS) is a representative conductive polymer widely utilized for biosensor surface engineering due to its mechanical flexibility and biocompatibility [62,63]. Recently, to address the chronic inflammatory responses resulting from the mechanical mismatch between rigid silicon-based probes and soft brain tissue, an all-organic PEDOT:PSS fiber microelectrode developed without thermal processing has been reported [64]. Phosphoric acid treatment selectively eliminated the PSS binder network and produced a porous fiber structure. Infiltration with ethylene glycol then enhanced the mechanical compliance of the fibers, yielding a low Young’s modulus (50.4 kPa) and a high fracture strain (8.7%). This structurally engineered fiber enabled stable, isolated recording of single-neuron spikes in the mouse hippocampus, and histological analysis further demonstrated its potential for chronic applications. Beyond PEDOT:PSS, conductive hydrogels have emerged as next-generation bioelectronic interfaces by integrating the electrical properties of conductive materials with the mechanical compatibility of hydrogels for soft biological tissues [65,66,67]. Their ability to simultaneously support electronic and ionic transport enables biointerfaces with signal transmission characteristics similar to those of biological tissues [68,69]. For example, since the hydrophobicity of conventional PEDOT:PSS limits its integration within the native aqueous environment of hydrophilic hydrogels, a hydrophilic self-doped conducting polymer (S-PEDOT) was developed by introducing sulfonic-acid side groups onto the monomer, enabling its homogeneous dispersion within the hydrogel matrix without forming aggregated PEDOT hard domains [70]. The resulting homogeneous network exhibited a high toughness (620 kJ m^−3^) and softness (10.5 kPa), overcoming the conventional toughness–softness trade-off. Furthermore, the hydrophilic nature of S-PEDOT enabled reversible drying and reswelling, providing improved reusability and handling convenience. As shown in Figure 2c, soft and stretchable conductive hydrogels improved the mechanical compatibility of implantable electronics with biological tissue by closely matching the modulus of the surrounding neural tissue [71]. This was achieved by blending a nontoxic plasticizing dopant into PEDOT:PSS, which induced phase separation into interconnected, highly conductive PEDOT-rich nanofibrils within a soft PSS-rich matrix. Upon hydration, the matrix swelled to form a stable hydrogel. By conformally adapting to the tissue surface, the resulting tissue–electrode interface reduced mechanical disparity, thereby improving electrical coupling and reducing chronic inflammatory responses during long-term implantation. PEDOT is also doped with specific carriers for drug delivery, though this approach is limited by the requirement for negatively charged drugs and insufficient loading capacity for meaningful effect [72]. To address this, PEDOT doped with mesoporous sulfonated silica nanoparticles (SNPs) has been reported to incorporate neurotransmitters such as glutamate and gamma-aminobutyric acid (GABA), which can be electrically triggered to release repeatedly. These drug-loaded SNPs were electropolymerized into the PEDOT film, and since SNPs are non-conductive, they insulate the loaded drug from strong electric potential, improving its stability during loading and stimulation. Release is triggered by electrochemical reduction of the PEDOT film; this platform was implanted using a mechanically flexible and biocompatible substrate, Parylene C, in the rat barrel cortex in vivo. Beyond PEDOT, another representative conductive polymer, polypyrrole (PPy), has been explored due to its stable conductivity across physiological pH ranges and biocompatibility. In one study, however, since direct deposition of PPy onto a hydrophobic substrate is difficult, sulfonated polyaniline (SP) was used as a pre-coating layer on the substrate, introducing hydrophilicity that enabled a more uniform deposition of PPy [73]. The sulfonic groups on SP introduced a negatively charged surface that electrostatically attracted the positively charged pyrrole monomers, leading to their uniform adsorption. This improved surface uniformity generated a spatially homogeneous intrinsic electric field, enabling modulation of the cell membrane potential and enhancing axonal elongation in Pheochromocytoma 12 (PC12) cells upon electrical stimulation. Overall, these conductive biointerface strategies show that balancing electrical conductivity with mechanical flexibility and biocompatibility is essential for maintaining stable signal transduction over prolonged implantation periods while providing opportunities for functions such as electrically regulated drug delivery.

### 2.4. Antifouling and Passivation Engineering

The long-term stability of biosensing systems is often limited by the nonspecific adsorption of interfering molecules, particularly in complex biological matrices such as blood [74]. For instance, an aptamer-based biosensor for the detection of serotonin was developed, in which a self-assembled monolayer (SAM) of thiolated DNA aptamers was immobilized onto an Au electrode [75]. To minimize undesired signals and enhanced selectivity, 6-mercapto-1-hexanol (MCH) was employed as a backfiller on the regions of the electrode surface not covered by the aptamers. This backfilling strategy effectively passivated the exposed Au surface, suppressing non-specific adsorption of interfering species and reducing the noise signal during electrochemical measurement. Beyond backfilling strategies involving molecular recognition layers, the intrinsic surface charge of a nanostructured electrode can itself provide selective antifouling without any recognition element. For example, mesoporous carbon (CMK-3) was functionalized with carboxyl groups to create a negatively charged surface, where electric double layer overlap within the nanoscale pores enabled charge-selective ion transport [76]. This electrostatic gating facilitated the entry of oppositely charged species while excluding ions with the same charge, thereby enhancing the transport of positively charged dopamine and suppressing the penetration of negatively charged ascorbic acid (AA). In addition, hydrophilic polymer coatings such as polyethylene glycol (PEG) form a hydrated interface that reduces hydrophobic interactions with surrounding proteins, resulting in reduced nonspecific protein adsorption [77]. A multiplex aptamer-functionalized microelectrode array was developed for the simultaneous detection of neurotransmitters (Figure 2d) [78]. Following aptamer immobilization on spatially separated electrodes, thiol-PEG was employed to backfill the unoccupied regions of the electrode surface. This hydrophilic thiol-PEG SAM prevented the adsorption of nonspecific proteins, enabling the detection of analytes in complex samples with high specificity. The limit of detection (LOD) measured in serum closely matched that obtained in buffer, demonstrating the applicability of the antifouling strategy to complex biological matrices. However, traditional PEG coatings form brush-like, 2D barriers that hinder target molecules from reaching the sensing probe, promoting the development of three-dimensional (3D) hydrogel architectures. A tetra-PEG hydrogel coating has been reported, formed by cross-linking four-arm PEG-thiol and four-arm PEG-maleimide via thiol-Michael addition [79]. In this homogeneous hydrogel network, the ether groups along the PEG backbone hydrogen-bond with water, generating a hydrated PEG-water layer that prevents nonspecific protein adsorption and preserves ionic pathways to the electrode, thereby minimizing fouling and maintaining signal fidelity. In addition, the porous PEG network enables size-selective transport, as the diameter of glycoproteins (200–500 kDa) is larger than the pore diameter of the gel (30.9 ± 0.5 nm), while preserving small-molecule transport. Under identical conditions using fouling agents such as bovine serum albumin (BSA) and whole blood, the resulting hydrogel-coated electrode retained distinct redox signals, while the bare electrode exhibited a substantial decrease in signal, demonstrating the antifouling capability of the network. Beyond PEG-based hydrogel, zwitterionic hydrogels have emerged as promising antifouling interfaces due to their ability to establish a more robust and stable hydration layer, which can sustain antifouling activity across a broader temperature range and for longer durations than conventional PEG-based coatings [80]. In one study, a poly(sulfobetaine) methacrylate hydrogel coating was applied to a depth probe implanted into the rat brainstem [81]. The zwitterionic hydrogel-coated probe maintained a stable impedance over 8 weeks and enabled single-neuron spike recordings for 4–5 weeks post-implantation, whereas uncoated probes exhibited a significant decline in recording quality within the first 2 weeks. As a result, backfilling, charge-selective, and hydration-based antifouling layers suppress nonspecific adsorption through distinct mechanisms, offering complementary approaches to enhance the long-term reliability of implantable biosensors in complex biological environments. Table 1 provides an overview of the representative surface engineering strategies discussed in this section, including nanostructured interface engineering, molecular recognition engineering, conductive biointerface engineering, and antifouling and passivation engineering, along with their fundamental principles and major advatages.

**Figure 2:**
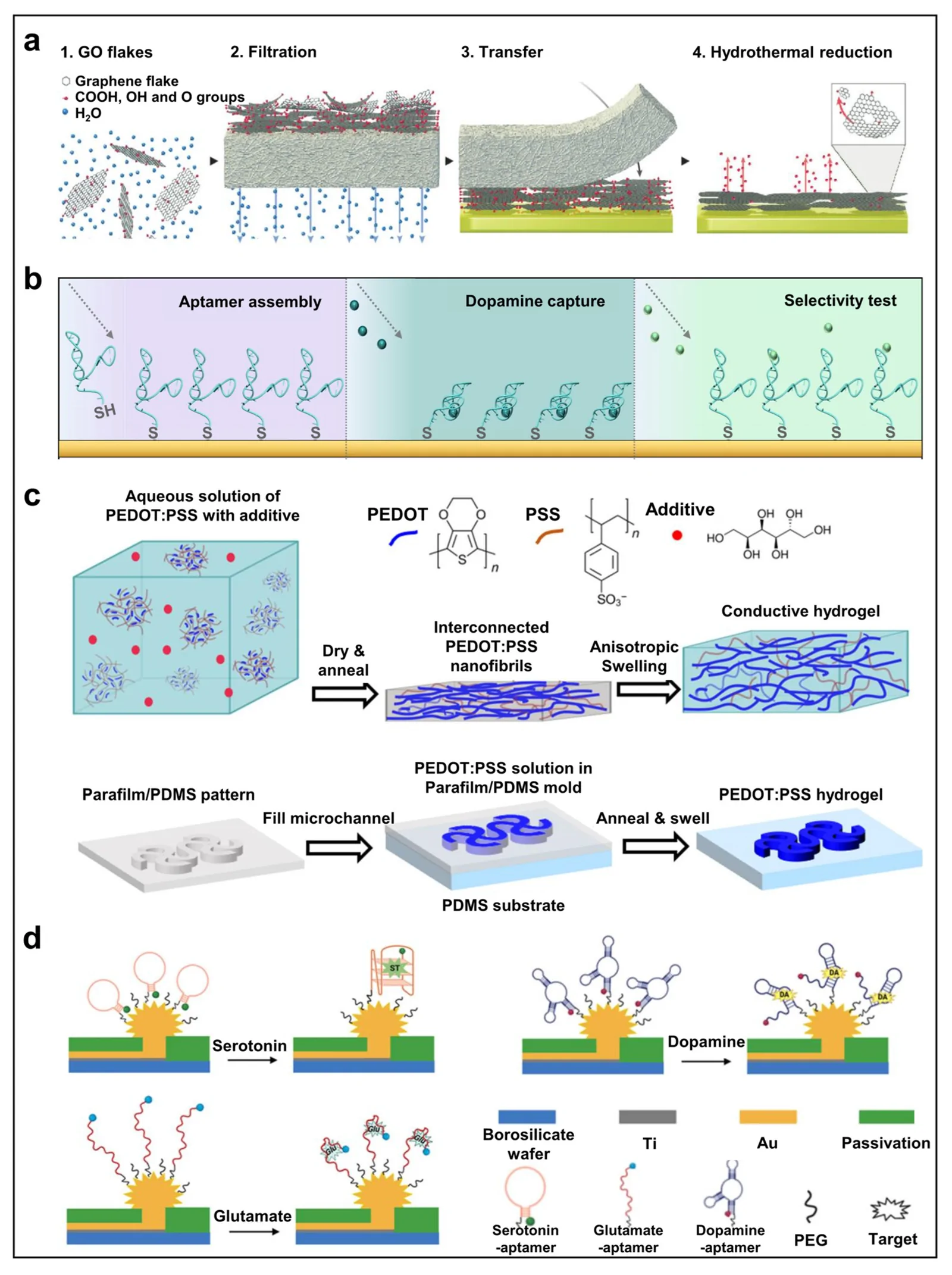
(**a**) Schematic illustration of nanoporous graphene-based thin films formed by stacking hydrothermally reduced graphene oxide flakes into a nanoporous network. Reproduced with permission from Ref. [39]. Copyright © 2024 The Author(s). (**b**) Schematic illustration of dopamine aptamer assembly and target-induced conformational changes. Reproduced with permission from Ref. [58]. Copyright © 2023 The Authors. Published by American Chemical Society (Washington, DC, USA). (**c**) Schematic image of the fabrication of PEDOT:PSS conductive hydrogel through the incorporation of a nontoxic plasticizing dopant. Reproduced with permission from Ref. [71]. Copyright © 2025 The American Association for the Advancement of Science. (**d**) Schematic image of a multiplexed electrochemical aptasensor for simultaneous detection of serotonin, glutamate, and dopamine using a hydrophilic thiol-PEG SAM. Reproduced with permission from Ref. [78]. Copyright © 2025 The Authors. Published by Elsevier B.V. (Amsterdam, The Netherlands).

**Table 1:** Classification and summary of surface engineering strategies for biosensors.

Surface Engineering Strategy	Category	Core Principle	Advantage/Effect	Ref.
Nanostructured interface engineering	MXene	Synergetic effect of MXene’s conductivity/ion accessibility and AuNP’s electrocatalytic activity	Increase in active-site density, conductivity, and catalytic activity	[31]
	MOF	Conversion of dopamine oxidation-induced faradaic current into a gating signal at the MOF channel	Spike-width broadening via synaptic facilitation in aqueous conditions	[35]
	ZIF-8	Size-matched physical encapsulation of the ionophore within ZIF-8 cavities and Zn-N coordination	Long-term stability of ion sensing in rat brain	[36]
		Locking of AuNPs within amine-functionalized ZIF-8 pores via in situ reduction	Promotion of dopamine adsorption and electron transfer	[37]
	COF	Conformational locking of the dopamine oxidation intermediate by COF nanopores	Stable real-time dopamine monitoring in a Parkinson’s disease model mouse	[38]
	Carbon	Hydrothermal stacking of reduced graphene oxide flakes into a nanoporous network	High capacitance and robust mechanical stability	[39]
		Oxygen plasma-induced CNT debundling via oxygen-containing functional groups, and DMSO-induced uniform Au nucleation via S-Au interaction	Uniform AuNP decoration with reduced SERS-intensity variation and improved reproducibility	[41]
Molecular recognition engineering	Antibody	Electric-field-driven rotation of dipolar IgG via opposite Fab/Fc surface charge	Higher antibody–antigen binding probability, boosting detection sensitivity in standard plasma	[46]
		Pepsin-mediated cleavage of IgG into Fab fragments, shortening the probe layer to fit within the Debye length	Increased sensitivity via higher receptor density, validated for NfL detection in TBI patient plasma	[49]
	Streptavidin–biotin	Pre-assembly of streptavidin-quantum dot and biotinylated-antibody probes via high-affinity streptavidin–biotin binding	Prevention of probe dissociation and antibody exchange, enabling multiplexed detection	[53]
	Aptamer	Target-induced conformational change of aptamers immobilized on nanopores, altering local charge density and ion permeability	Selective discrimination of dopamine from structurally similar molecules	[58]
	MIP	Polymerization of monomers around target templates to form complementary binding cavities	Simultaneous NGF/proNGF discrimination based on current reduction upon target binding	[61]
Conductive biointerface engineering	PEDOT:PSS	Phosphoric acid removal of the PSS binder network to form a porous fiber, followed by ethylene glycol infiltration, enhancing chain compliance	Low Young’s modulus and high fracture strain, enabling stable single-neuron recording in mouse hippocampus	[64]
	Conductive hydrogel	Sulfonic-acid self-doping of the EDOT monomer, enabling homogeneous S-PEDOT dispersion within the hydrogel matrix	High toughness and softness, overcoming the conventional toughness–softness trade-off	[70]
		Blending of a nontoxic plasticizing dopant into PEDOT:PSS, inducing phase separation into conductive PEDOT-rich nanofibrils within a swellable PSS-rich matrix	Tissue-matching modulus and conformal adaptation, improving electrical coupling and reducing chronic inflammation	[71]
	PEDOT-SNP	Electropolymerization of neurotransmitter-loaded, non-conductive SNPs into the PEDOT film, insulating the cargo from electric potential	Stable, repeatable, electrically triggered Glutamate/GABA release for reversible neuromodulation in the rat barrel cortex	[72]
	PPy	SP pre-coating that electrostatically attracts positively charged pyrrole monomers, enabling uniform PPy deposition	Spatially homogeneous electric field enhancing axonal elongation in PC12 cells upon electrical stimulation	[73]
Antifouling and Passivation engineering	MCH	Thiol-based backfilling of MCH, blocking non-specifically adsorbed species on the exposed Au surface	Suppressed non-specific adsorption and reduced background noise, enabling selective serotonin detection	[75]
	CMK-3	Carboxyl functionalization of CMK-3, creating a negatively charged surface and enabling charge-selective ion transport	Enhanced transport of positively charged dopamine while suppressing negatively charged AA	[76]
	PEG	Thiol-PEG backfilling of unoccupied electrode regions, forming a hydrated interface that reduces hydrophobic protein interactions	Prevention of nonspecific protein adsorption, with a serum LOD closely matching that obtained in buffer	[78]
		Cross-linking of four-arm PEG-thiol and four-arm PEG-maleimide into a 3D hydrogel network whose pore size excludes glycoproteins while preserving ionic pathways	Size-selective antifouling that maintains distinct redox signals under BSA and whole-blood fouling conditions	[79]
	Zwitterion	Poly(sulfobetaine) methacrylate coating establishing a stable hydration layer, enabling sustained antifouling activity	Stable impedance and single-neuron spike recording for 4–5 weeks post-implantation in rat brainstem	[81]

## 3. Surface-Engineered Biosensors for Extracellular Soluble Neural Biomarker Detection

Neural biomarkers serve as molecular indicators reflecting the state of the nervous system and provide important information for the detection and prognostic assessment of various neurological disorders [82,83]. In particular, neurodegenerative disorders involve progressive molecular alterations before the onset of symptoms. Therefore, early and accurate detection of relevant biomarkers is critical for identifying neurological disorders at earlier stages than conventional imaging-based methods such as computed tomography, magnetic resonance imaging and magnetic resonance angiography. However, these biomarkers are present at extremely low concentrations in biological samples and are affected by various interfering substances in real samples, requiring surface engineering strategies to develop neural biosensors with high sensitivity and selectivity [84,85]. This chapter discusses representative neural biomarkers, including Aβ, dopamine, and miRNA, and introduces surface-engineered biosensors for extracellular detection of these biomarkers, with a focus on strategies to improve biosensing performance.

Aβ is generated through the proteolytic cleavage of amyloid precursor protein. It is a representative biomarker of Alzheimer’s disease (AD) that readily aggregates to form oligomers and amyloid plaques. The accumulation of Aβ is considered one of the core pathological features of AD. Clinically, Aβ serves as a biomarker for assessing the presence of amyloid pathology associated with AD in patients [86,87]. Accordingly, various surface-engineered biosensors have been developed for the early and accurate detection of Aβ. One research group reported a dual prism-assisted solution-immersed silicon (DP-SIS) optical neural biosensor (Figure 3a) [88]. Optical biosensors offer the advantage of label-free and real-time detection by directly measuring changes in the refractive index and optical thickness of the sensor surface induced by biomolecular binding. Taking advantage of these characteristics, the authors developed an optical biosensor for the label-free detection of Aβ1-42. In this platform, amine groups were introduced onto the silicon surface through 3-aminopropyltriethoxysilane (APTES) treatment, and an Aβ1-42-specific antibody was immobilized on the sensor surface via streptavidin–biotin interactions. In addition, a buffer containing phosphate-buffered saline (PBS) and BSA was introduced to block residual active sites, thereby suppressing nonspecific adsorption in biological samples and improving the signal-to-noise ratio. Upon binding of Aβ to the antibody, changes in the thickness and optical properties of the sensor surface occur, and the resulting changes in the polarization characteristics of the reflected light were monitored in real time to detect Aβ. Subsequently, the effects of nonspecific signals caused by serum components, including albumin and immunoglobulins, were evaluated to assess the applicability of the biosensor to biological samples. Aβ1-42 was spiked into Aβ-depleted serum at concentrations ranging from 0.1 pg/mL to 10 ng/mL. Even after correcting for the background signal caused by interfering substances in serum, a detectable signal was observed at a low concentration of 0.1 pg/mL, with a signal-to-noise ratio of 2.36. Consequently, the DP-SIS optical neural biosensor achieved an LOD of 0.04 fg/mL in the defined buffer and 12.0 fg/mL in human serum. A graphene electrolyte-gated field-effect transistor (G-EGT)-based neural biosensor was also developed for the detection of Aβ in neuron-derived exosomes isolated from blood [89]. Here, AuNPs were deposited onto the surface of reduced graphene oxide (rGO) to increase the surface area, thereby enabling the immobilization of a greater number of anti-Aβ antibodies. Subsequently, BSA and Tween-20 were introduced as a dual-blocking surface engineering strategy to suppress nonspecific binding of anti-Aβ antibodies. From the results, this neural biosensor achieved ultrasensitive detection of Aβ by monitoring changes in the electrical characteristics of the G-EGT induced by biomarker binding, with an LOD of 447 ag/mL. In another approach, Cys-Aβ was immobilized on the surface of AuNPs, and the target Aβ was detected by measuring changes in the extinction spectrum resulting from surface plasmon resonance (SPR) coupling between the AuNPs, triggered by Aβ aggregation [90]. However, beyond simply determining the presence or concentration of Aβ, characterizing its various aggregation states, ranging from monomers to oligomers and fibrils, is important for understanding the pathological characteristics of the disease. Therefore, biosensors capable of characterizing the formation of amyloid aggregates or their properties at the single-molecule level have been reported, extending beyond conventional optical and electrochemical biosensors [91,92,93]. As one example, a nanopore biosensor has been developed using a biological nanopore to monitor the aggregation kinetics of β-amyloid peptides in real time [91]. This approach detects changes in electrical signals generated when Aβ peptides pass through or interact with the nanopore, enabling the dynamic changes occurring during Aβ aggregation to be monitored at the molecular level. Such nanopore-based sensing platforms can be applied to the single-molecule characterization of various forms of protein aggregates. In particular, biological nanopores can also be used for protein sequencing, providing opportunities to elucidate the structural properties and heterogeneity proteins that are prone to aggregation [92,93]. In addition to Aβ accumulation, tau protein is a major biomarker that reflects pathological changes associated with AD, with their plasma concentrations being closely associated with AD pathology. In clinical settings, these biomarkers have attracted significant attention as indicators for determining the presence of AD pathology in patients with cognitive impairment and predicting subsequent cognitive decline and progression to AD dementia in individuals with mild cognitive impairment [94,95,96]. Conventional detection methods of tau proteins, such as enzyme-linked immunosorbent assay (ELISA) and mass spectrometry, have limitations in detecting tau protein present at low concentrations in blood, thereby requiring signal amplification strategies to improve detection sensitivity [97]. Recently, a wearable electrochemical sensing patch incorporating various surface engineering strategies was developed for the detection of tau protein in interstitial fluid. The nanogold-modified vertical graphene (VG@Au) structure, fabricated by depositing a gold layer onto a VG electrode, enhanced electron transfer and antibody immobilization efficiency, thereby improving sensor sensitivity [98]. In addition, a polydopamine (PDA) layer formed through dopamine self-polymerization was functionalized onto the transistor surface, where the electrostatic properties of PDA facilitated the oriented immobilization of antibodies through interactions between its amine groups and the Fc region of the antibodies [99]. This well-organized antibody orientation maximized the exposure of the antigen-binding Fab region, resulting in improved tau-binding efficiency and reduced nonspecific binding. In contrast, studies have extended the application of polymer-based surface engineering strategies beyond antibody immobilization to facilitate charge transfer and enhance signal amplification. For example, to enable point-of-care detection of phosphorylated tau protein 181 (p-tau181), an electrochemical neural biosensor was developed by integrating a polyphenol red–PPy (PPhR-PPy)-based nano MIP (nMIP) with a highly porous gold (hPG) electrode (Figure 3b) [100]. An hPG layer was first deposited onto a printed circuit board (PCB)-based electrode, followed by electropolymerization of a PPhR-PPy layer using p-tau181 as the template molecule. Subsequent oxalic acid treatment removed the template, generating molecularly imprinted cavities for selective recognition of p-tau181. The resulting polymer layer functioned both as a recognition receptor and an electrochemical signal transduction layer. The hPG increased the electrode surface area, facilitating target access and electrochemical reactions, while the redox activity of PPhR enabled label-free signal generation. The incorporation of PPy further improved electron transfer and the mechanical stability of the polymer layer, thereby enhancing sensor performance. Binding of p-tau181 to the imprinted cavities altered the electrochemical environment and charge transfer characteristics of the electrode, which was monitored by differential pulse voltammetry (DPV). Compared with the hPG/PPhR sensor, the hPG/PPhR-PPy sensor showed approximately twofold higher sensitivity, attributed to the improved electrochemical properties resulting from PPy incorporation. The biosensor achieved a low LOD of 498 fg/mL and a linear quantification range of 1–16 pg/mL (R^2^ = 0.982) in undiluted human serum. It also showed a higher response to p-tau181 than tau441, confirming its selective recognition capability. In a clinical evaluation using plasma samples from patients with AD and healthy controls, an area under the curve (AUC) of 0.84 was obtained, compared with 0.81 for the hPG/PPhR sensor. Furthermore, measurements using 400 independently fabricated biosensors showed consistent electrochemical signals, demonstrating high reproducibility.

Similar to Aβ and phosphorylated tau proteins, which serve as major pathological biomarkers of AD, α-synuclein (α-syn) is a representative protein biomarker of PD that reflects the pathological features of the disease. Because α-syn begins to accumulate in the early stages of the disease, detecting it before irreversible neuronal damage occurs is critical [101]. Accordingly, an aptamer-based organic electrolyte-gated field-effect transistor (FET)-based neural biosensor capable of selectively detecting α-syn monomers through noninvasive saliva-based analysis has been reported [102]. However, the toxicity and aggregation associated with disease progression are primarily induced by α-syn oligomers and fibrils. Therefore, recent studies have focused on the development of biosensors for the detection of α-syn oligomers. One such example is AuNP/laser-induced graphene (AuNP/LIG)-based electrochemical immunosensor developed for the detection of α-syn in blood samples [103]. This neural biosensor was constructed using an LIG electrode, onto which AuNPs were electrochemically deposited, followed by the immobilization of an α-syn-specific antibody to form a target-recognition interface. The AuNPs deposited on the LIG surface formed a nanostructured interface that enhanced the electrochemical properties of the electrode while increasing the surface area available for antibody immobilization. Subsequently, BSA was used to reduce the nonspecific adsorption of biomolecules in blood samples. These surface modifications enabled the effective detection of electrochemical changes at the interface induced by antigen–antibody binding. Upon binding of α-syn to the immobilized antibody, the charge-transfer properties at the electrode–electrolyte interface were altered. These changes were monitored using electrochemical impedance spectroscopy (EIS) by measuring variations in the charge-transfer resistance (Rct). The neural biosensor exhibited a linear response over a concentration range of 0.01–100 ng/mL, with an LOD of 0.237 pg/mL. To evaluate its practical applicability, human blood samples from four patients with PD and four healthy controls were analyzed. The mean change in Rct was 1.488% in the healthy control group, whereas a higher change of 8.38% was observed in the PD group. Thus, the AuNP/LIG-based electrochemical immunosensor demonstrated its ability to detect α-syn in actual blood samples and distinguish between PD patients and healthy controls, highlighting its potential as a sensing platform for the blood-based detection of α-syn. Moreover, a nanoplasmonic neural biosensor was developed to distinguish different α-syn aggregation states. The biosensor selectively captured α-syn using specific antibodies immobilized on a nanoplasmonic metasurface and analyzed signal changes associated with α-syn structural properties in the infrared region. It quantitatively distinguished α-syn oligomers and fibrils in complex mixtures, demonstrating its potential for detecting pathological aggregation states [104]. The abnormal aggregation of α-syn progressively leads to the loss of dopaminergic neurons in the substantia nigra, resulting in reduced dopamine production and release, a characteristic pathological feature of PD [105,106]. Although dopamine has not yet been established as a definitive clinical diagnostic biomarker for PD, quantification of dopamine levels may serve as a useful tool for identifying neurological disorders [107]. For instance, an Fe-incorporated hierarchical porous seaweed carbon (Fe-HPSW-C) sensing electrode was fabricated by growing an Fe-based MOF, MIL-53 (Fe), film on a carbonized seaweed carbon substrate [108]. The resulting porous structure enhanced dopamine adsorption and electrochemical signals. However, since such nanomaterial-based biosensors rely on dopamine oxidation, selective recognition strategies are required to minimize interference from other redox-active molecules in complex biological environments. As a result, recent studies have explored the use of biomolecules for selective target recognition. For example, dopamine-specific aptamers were immobilized onto a nanoplasmonic electrode to enable the detection of dopamine in untreated whole blood by monitoring plasmonic signal changes induced by dopamine binding [109]. Another study developed a ratiometric electrochemical neural biosensor by modifying an electrode with a Zr/Ni bimetallic MOF containing electrochemically active 1,1′-ferrocenedicarboxylic acid (Fc) (Zr/Ni-FcDA MOF) as an organic ligand, followed by immobilization of a dopamine-specific aptamer [110]. The Fc oxidation signal served as an internal reference, and dopamine was quantified from the ratio of the dopamine and Fc signals, thereby reducing signal variations caused by electrode conditions and enabling stable quantification. The incorporation of MXene further enhanced electron transfer and provided a large surface area. The dopamine-specific aptamer enabled selective recognition, with a markedly higher signal ratio for 20 μM dopamine than for the interfering substances uric acid and serotonin at 200 μM. As the dopamine concentration increased, its oxidation peak increased, whereas the Fc peak remained nearly constant, which confirmed that the signal ratio varied with the dopamine concentration. The neural biosensor showed excellent linearity over a concentration range of 0.1–70 μM in PBS (R^2^ = 0.9982) with an LOD of 0.07 μM. Reproducibility evaluation using six independently fabricated electrodes from different batches showed a relative standard deviation of 3.6%, and 90.80% of the initial signal was retained after 6 days, demonstrating good stability. In addition, human serum spiked with 10, 20, and 30 μM dopamine showed recovery rates of 98.90%, 100.5%, and 98.67%, respectively, with standard deviations of 1.8–4.8%, indicating good quantitative accuracy and precision in complex biological samples. Thus, the Zr/Ni-FcDA MOF incorporated with MXene composite enhanced the electrode conductivity, while the dopamine-specific aptamer provided selective molecular recognition, enabling sensitive and stable dopamine detection. More recently, an electrochemical neural biosensor for dopamine detection was developed by modifying the electrode surface with a nanocomposite of rGO and copper ferrite. The integration of copper ferrite with rGO enhanced the electrochemical properties of the electrode and promoted dopamine adsorption and oxidation, thereby improving the sensitivity and enabling selective dopamine detection [111]. MicroRNAs (miRNAs) are short non-coding RNAs that regulate gene expression. In particular, altered expression of specific miRNAs in AD and PD has been associated with pathways involved in Aβ and tau protein metabolism, as well as α-syn and dopaminergic neuronal function. Accordingly, miRNAs have attracted considerable attention as biomarkers for screening neurological disorders using body fluids such as blood and serum. Therefore, various surface-engineered biosensors have been developed for their detection [112,113,114]. Among these studies, an SPR imaging (SPRi) neural biosensor was developed using a tetrahedral DNA nanostructure-based surface engineering strategy to improve probe orientation and hybridization efficiency, combined with CRISPR/Cas-based signal amplification [115]. The SPRi neural biosensor enabled detection of miRNAs related to neurological disorders by monitoring SPR signal changes induced by target miRNA hybridization. Meanwhile, various strategies using functional polymers have been reported to improve the stability of probe immobilization and the electrochemical performance of electrodes [116,117]. One study developed an electrochemical neural biosensor for the detection of salivary miRNAs associated with mild TBI (mTBI) using a functional polymer and a COF (Figure 3c) [117]. After forming a COF layer on the electrode surface, streptavidin was covalently immobilized via (3-glycidoxypropyl)trimethoxysilane (GPTMS). Subsequently, methylene blue (MB)- and biotin-labeled anti-miRNA probes were stably immobilized. The large surface area of the COF enabled the immobilization of a high density of anti-miRNA probes. In addition, Nafion, introduced as a conductive polymer layer, facilitated cation transport and improved electrode conductivity, while another conductive polymer layer, PEDOT, reduced the overpotential of the MB redox reaction, thereby enhancing the electrochemical signal and improving the conductivity and sensitivity of the sensor. The neural biosensor detected the target miRNAs through a turn-off mechanism, in which hybridization of the target miRNAs with the immobilized anti-miRNA probes increased the distance between MB and the electrode, thereby reducing electron-transfer efficiency and decreasing the current signal. As a result, an LOD of 0.4–1 nM was achieved using miRNA standard solutions. In addition, the neural biosensor exhibited selectivity toward the target miRNAs, as no significant cross-reactivity with nonspecific miRNAs was observed in artificial saliva, and maintained stable performance for up to 60 days after fabrication. In clinical validation, saliva samples from 15 patients with mTBI and 15 healthy controls were analyzed. This neural biosensor showed 100% sensitivity and specificity for mTBI discrimination, in good agreement with the polymerase chain reaction (PCR) results. Notably, polymers have been utilized not only as surface coating materials to improve biomolecule immobilization efficiency and biocompatibility but also as miRNA-sensing platforms themselves, such as hydrogel-based systems. These polymeric neural biosensors utilize hydrogels containing catalytic hairpin assembly-based probes, enabling enzyme-free fluorescence signal amplification under isothermal conditions for miRNA detection [118].

Beyond the representative neural biomarkers discussed in this section—Aβ, tau protein, α-syn, dopamine, and miRNA—NfL and glial fibrillary acidic protein (GFAP) have also emerged as important biomarkers for assessing neurological disorders [119,120]. NfL is a structural protein of neuronal axons, and increased NfL levels in the body are associated with axonal injury and neurodegeneration. GFAP is a protein predominantly expressed in astrocytes, and increased GFAP levels are associated with astrocytic activation and an increased rate of cognitive decline [120]. Therefore, NfL and GFAP provide information on neuronal and glial alterations, respectively, thereby expanding the range of biomarkers associated with neurological disorders. Neural biosensors incorporating diverse surface engineering strategies enable the sensitive and selective detection of these neural biomarkers, thereby contributing to the early detection of neurological disorders. As surface-engineered biosensors continue to advance, recent studies have expanded beyond the detection of extracellular neural biomarkers toward the detection and monitoring of biomarkers at the cellular level.

**Figure 3:**
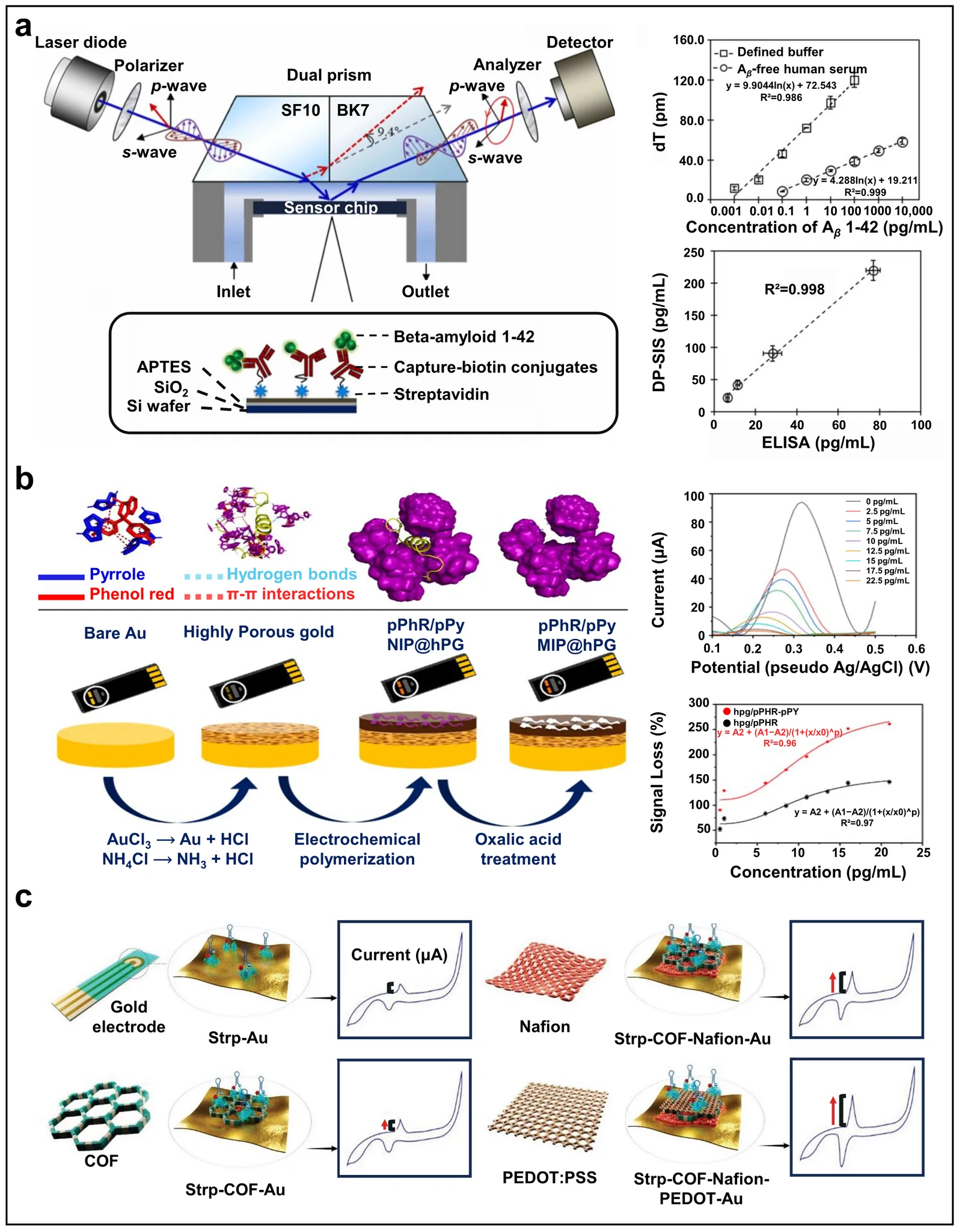
(**a**) Schematic illustration of the DP-SIS optical neural biosensor for Aβ detection. Sensing signals at different Aβ concentrations and comparison of the DP-SIS optical neural biosensor with ELISA for validation. Reproduced with permission from Ref. [88]. Copyright © 2023 The Authors. Published by Elsevier B.V. (**b**) Scheme of nMIP-based neural biosensor for p-tau181 detection. DPV signals at various p-tau181 concentrations and the corresponding calibration curve. Reproduced with permission from Ref. [100]. Copyright © 2025 The Authors. Published by American Chemical Society. (**c**) Schematic diagram of surface engineering strategies using COF, Nafion, and PEDOT:PSS on gold electrodes for enhanced electrochemical miRNA detection. Reproduced with permission from Ref. [117]. Copyright © 2025 The Author(s). Small published by Wiley-VCH GmbH (Hoboken, NJ, USA).

## 4. Surface-Engineered Biosensors for Neural Biomarker Detection at the Cellular Level

Although surface-engineered neural biosensors have been developed to detect soluble neural biomarkers, such as dopamine and Aβ, with high sensitivity and selectivity, these approaches alone are insufficient to fully capture the dynamic physiological changes occurring at the cellular level. Therefore, real-time monitoring from living cells is essential for the accurate and rapid detection of neurological disorders and a more comprehensive understanding of their underlying pathophysiology [121,122]. To this end, recent studies have been increasingly focused on integrating advanced surface engineering strategies into biosensor platforms to improve the stability of the biointerface with living cells and enable real-time monitoring of neural biomarkers at the cellular level. In this chapter, we highlight representative surface-engineered neural biosensors capable of sensing neural biomarkers at the cellular level. Importantly, at the cellular level, most studies have focused on dopamine, due to its distinct redox properties and easily induced exocytotic release from dopaminergic cell models. In contrast, surface-engineered biosensors targeting other clinically relevant biomarkers, such as NfL and GFAP, remain relatively limited. This imbalance indicates a current research gap and suggests the need to expand biosensor development beyond dopamine-based sensing systems.

Nanostructured interface engineering strategies provide an efficient electrode–cell interface by facilitating charge transfer and enhancing electrochemical signal transduction [123,124]. Recent advances have evolved toward integrating metal nanostructures with carbon-based materials or functional molecules to simultaneously enhance signal amplification and sensing selectivity, thereby enabling real-time monitoring of neurotransmitters released from living neural cells. In one study, a graphene oxide (GO)-wrapped hierarchical Au nanopillar hybrid structure was fabricated on the electrode surface to provide a large electroactive surface area and efficient charge transfer at the electrode–cell interface [125]. The GO layer enhanced dopamine adsorption through π–π interactions and suppressed electrode fouling, while the Au nanopillars promoted electrocatalytic activity and signal amplification. This synergistic hybrid structure achieved a 2.16-fold lower ΔEp than the control, indicating faster electron transfer, and lowered the LOD from 9.98 μM (control) to 0.0295 μM. These enhancements enabled the real-time, non-destructive detection of dopamine released from human induced pluripotent stem cell (iPSC)-derived dopaminergic neurons. In another study, a functionally collaborative nanostructure composed of ZIF-8-coated zinc oxide (ZnO) nanowires grown in situ on rGO was developed to directly monitor dopamine exocytosis from living cells [126]. Unlike conventional carbon fiber electrodes, which typically measure exocytotic events from only a single cell in real time, this sensor enabled simultaneous, continuous monitoring of individual exocytotic events from one to tens of living PC12 cells. In this hierarchical architecture, the ZIF-8 shell served as a solid-base nanocatalyst that selectively adsorbed and catalytically oxidized dopamine, while the rGO nanosheets facilitated rapid electron transport and the vertically aligned ZnO nanowires increased the effective sensing area by capturing exocytotic events from multiple directions. Due to the synergistic functions of these components, the microsensor achieved an ultralow LOD less than 1 pM with high selectivity toward dopamine, whereas the ZnO-on-rGO skeleton control exhibited an LOD above 1 μM. Recently, studies have also explored the use of metal clusters and supramolecular architectures to further enhance the molecular detection of neurotransmitters. For instance, a polyoxometalate (POM)-γ-cyclodextrin MOF interface, immobilized on a graphene electrode via a Nafion binder that ensured stable adhesion and antifouling properties, was engineered to provide electrocatalytic signal amplification for dopamine oxidation, enabling the selective and direct detection of dopamine released from neuroblastoma cells [127]. Using this platform, real-time dopamine secretion from K^+^-stimulated SH-SY5Y cells was quantitatively tracked at multiple time points, demonstrating time-dependent neurotransmitter release. Furthermore, hybrid nanocomposites integrating multiple conductive and catalytic components have also been developed to simultaneously enhance interfacial conductivity and electrocatalytic activity. In one study, a platinum (Pt) nanowire/MXene/porous carbon ternary nanocomposite was fabricated, in which the grain boundaries and undercoordinated surface atoms of the Pt nanowires provided abundant active sites for dopamine oxidation [128]. MXene nanosheets facilitated rapid electron transfer, while the porous carbon framework promoted dopamine adsorption at the electrode surface. This synergistic architecture, together with the specific affinity between the amine group of dopamine and the Pt surface, contributed to enhanced sensitivity and selectivity, enabling in situ monitoring of dopamine release from neural cells. In addition, a nickel cobaltite/multi-walled carbon nanotube (NC/MWCNT) flexible sensor has been reported for the detection of dopamine [129]. In the NC/MWCNT composite, NC provides the redox-active surface for dopamine oxidation, and density functional theory (DFT) calculations demonstrated the favorable binding between the oxygen atom of dopamine to the Co sites on the NC sheet. When MWCNT was positioned perpendicular to the NC sheet, the amount of charge transferred from dopamine to NC increased from 0.11 e^−^ to 0.16 e^−^, indicating that MWCNT strengthens this chemisorption. The NC/MWCNT-modified electrode also reduced charge transfer resistance (from the kilo-ohm to the ohm range), reflecting the enhanced conductivity gained through the incorporation of MWCNT. Stability (92% current retention over 20 measurements), inter-electrode reproducibility (five electrodes), and durability (84.9% current retention after 30 days) further supported the sensor’s reliability. Finally, integration onto a flexible substrate achieved detection of 1 nM dopamine in standard solution, and the sensor successfully detected dopamine released by sub-cultured SH-SY5Y human neural cells upon KCl-induced stimulation, confirming its applicability in real-time, cell-level dopamine monitoring.

Recently, molecular recognition engineering has evolved beyond simple molecular recognition toward establishing stable biointerfaces with living cells, enabling the long-term and selective real-time monitoring of neural biomarkers secreted from or expressed by cells [130,131]. Accordingly, this shift has led to the development of recognition strategies that enable reversible binding, which allows repeated real-time measurements without replacing the recognition probes. Conventional neurotransmitter biosensors have been limited due to the high binding affinity between probe and target, which makes it difficult to reuse the sensor, especially during continuous monitoring. To overcome this, a semiconducting CNT film FET was developed, capable of in situ electric resetting using a pH-driven recovery strategy (Figure 4a) [132]. A CG-rich, pH-sensitive sequence was incorporated into the aptamer probe, allowing local pH modulation via an on-chip palladium electrode by tuning its potential. Under acidic conditions, the aptamer switched into a triplex conformation, reducing its affinity and releasing the target, while reverting to neutral conditions recovered its original binding capability within about one minute. The sensor achieved an LOD of 10 fM with a detection range spanning eight orders of magnitude for dopamine, serotonin, histamine, and glutamate. For simultaneous multiplexed detection of these targets, four regions of a single chip were functionalized with target-specific aptamers and integrated with a polydimethylsiloxane (PDMS) microfluidic channel to suppress cross-activity, achieving stable responses over ten reuse cycles. The sensing platform was further demonstrated for real-time monitoring of neurotransmitter release from NGF-differentiated PC12 cells, confirming its applicability to measurements in a cellular environment. Beyond restoring probe reusability, molecular recognition has also been advanced to distinguish dopamine dynamics between the intracellular and extracellular microenvironments of a single living cell. For instance, a dual-nanopore biosensor was developed by integrating aptamer-based molecular recognition into a nanopore platform, enabling dopamine detection in both the intracellular and extracellular settings of single PC12 cells while avoiding membrane interference [133]. Integrating two nanopores within a single capillary enabled complete circuit formation inside the pipette without transmembrane electrical coupling. For intracellular sensing, the probe generated a current decrease and reached a plateau within 10 min, indicating the presence of intracellular dopamine. Upon Levodopa (L-DOPA) stimulation, the current decreased further, reflecting an increase in intracellular dopamine levels. For extracellular efflux measurement, a single cell was instead confined within a microwell chip, where the limited volume facilitated local accumulation of dopamine. Under normoxic conditions, PC12 cells showed minimal response, whereas under hypoxic conditions, a distinct current response was observed, consistent with hypoxia-induced dopamine secretion. While enhanced recognition specificity is important, stable long-term interfacing with living cells also requires antifouling and passivation strategies capable of suppressing nonspecific biological adsorption. In one study, lubricin (LUB), a glycoprotein containing adhesive globular domains at both its N- and C- termini, was introduced as an antifouling agent capable of forming a SAM on the surface (Figure 4b) [134]. The central mucin domain forms a hydrophilic hydration layer that creates a nanopore-like structure, allowing small target molecules, such as NGF, to diffuse through while selectively excluding larger interfering proteins such as BSA. This nanopore-like architecture therefore enables size-selective antifouling while maintaining accessibility to the target molecules. Under BSA conditions, the LUB-modified immunosensor achieved an LOD of 0.048 nM, compared with 0.95 nM for the unmodified sensor. To validate the platform in a physiologically relevant setting, NGF was encapsulated within gelatin methacryloyl (GelMA) hydrogels, which were co-cultured with PC12 cells. This model generated extracellular metabolic byproducts and additional biomolecules, providing a complex biological environment. NGF concentrations in the culture media were then measured at selected time points (days 1, 3, and 5) using the LUB-based electrochemical sensing platform and a conventional ELISA. The two methods showed no statistically significant differences, confirming the accuracy of the LUB-based sensing platform while demonstrating its ability to rapidly detect NGF compared with conventional ELISA. As an alternative to synthetic antifouling coatings, biomimetic passivation using native cell membrane components can reduce nonspecific adsorption in living systems. In one study, a carbon fiber microelectrode (CFE) was engineered as a multilayer biomimetic sensor for dopamine detection [135]. Chitosan was first electrodeposited as a biocompatible base layer, on which the brain cell membrane was coated via amide bonding and electrostatic interactions. A dopamine-specific cholesterol-conjugated aptamer was then anchored within the membrane coating via its cholesterol group. The antifouling performance of the membrane coating was confirmed under BSA exposure, with only a 3% decrease in current response compared with a 60% decrease for the bare CFE. To demonstrate its applicability to living cells, the resulting electrode was applied to PC12 cells subjected to lipopolysaccharide (LPS)-induced neuroinflammation as a model of PD pathology. Upon K^+^ stimulation, the electrode exhibited a rapid increase in current followed by a stable response, whereas the current decreased after 30 min of incubation with 2 μg/L LPS. These results demonstrated the sensitive and stable electrochemical response of the electrode in a living cell population.

Stable monitoring of neurotransmitters at the cellular level requires electrode interfaces that provide not only high electrical conductivity but also long-term interfacial stability and biocompatibility with living cells [136,137]. To meet these requirements, polymeric surface engineering strategies based on conductive polymers, particularly PEDOT:PSS, have been widely explored to enhance electrical coupling while establishing cell-friendly biointerfaces. A representative example of conductive biointerface engineering was reported using negatively charged PEDOT:PSS hydrogel cross-linked with positively charged conductive polymer monomers to form a non-toxic conductive scaffold [138]. The resulting hydrogels showed elastic moduli ranging from ~50 Pa to ~300 Pa, closely matching native tissue, while the PEDOT:PSS combined with polyindole-5-NH_2_ (PEDOT:PSS/PIn-5-NH_2_) was selected for its highest conductivity (3265 S/m). PEDOT:PSS/PIn-5-NH_2_ also exhibited enhanced biocompatibility and was then compared to a conventional 4-dodecylbenzenesulfonic acid (DBSA)-based hydrogel, which significantly reduced cell viability by more than half. AuNPs were electrodeposited onto the hydrogel, formed on a glassy carbon electrode (GCE), to enhance electrocatalytic performance, achieving an LOD of 0.01 μM, good reproducibility across five electrodes, and 86.11% signal retention after 9 days of storage in PBS. To extend this platform to living cells, the hydrogel was formed within a cylindrical reservoir on an indium tin oxide (ITO) electrode to construct a 3D biosensor, and PC12 cells were subsequently incubated on the surface. Upon stimulation, the sensor recorded current response to 10 mM K^+^, which approximately doubled when the concentration of K^+^ was 5 mM, reflecting concentration-dependent secretion. In addition, pre-treatment with the calcium channel blocker nifedipine significantly suppressed this response, indicating a calcium channel-dependent mechanism of dopamine release and demonstrating the capability of this platform for pharmacological validation. PPy has also been recognized as a conductive and biocompatible polymer capable of supporting living cell attachment and differentiation [139,140] while providing an electroactive scaffold for the development of nanostructured dopamine sensors. Accordingly, a GO-integrated PPy/Au nanocluster hybrid nanopattern array on ITO was developed via electrochemical deposition, forming a 3D nanopillar structure where GO facilitated the polymerization of pyrrole while promoting dopamine adsorption through π–π stacking and hydrogen bonding (Figure 4c) [141]. Au nanoclusters were further incorporated to catalyze dopamine oxidation and reduction, amplifying sensitivity by 2.35-fold and yielding a larger electroactive surface area, an LOD of 33.4 nM, and excellent reproducibility across six repeated measurements. To validate this platform biologically, SH-SY5Y cells were differentiated into mature dopaminergic neurons by introducing neurogenic factors, showing enhanced cell adhesion and increased cell growth on the sensor. In addition, the DPV signal increased linearly over 12 days (11.0 μA to 21.5 μA), closely matching the upregulation of dopaminergic markers such as tyrosine hydroxylase (TH) and dopamine transporter (DAT). The platform further tracked the full differentiation process from iPSCs through neural stem cells to mature dopaminergic neurons, showing a concentration-dependent signal increase with L-DOPA under KCl treatment. When tested against diverse cell types, including fibroblasts, endothelial cells, several cancer cell lines, iPSCs, and neural progenitor cells, the signal was detected exclusively from dopaminergic neurons, demonstrating the ability to nondestructively and noninvasively discriminate functional dopamine-secreting cells. These findings support its potential for developing effective stem cell-based therapeutics.

These advances demonstrate that surface engineering strategies have enabled neural biosensors to progress from highly sensitive molecular detection toward stable, long-term, and biocompatible monitoring of dynamic neural activities at the cellular level. More recently, this research focus has been extended toward increasingly complex biological models, where neural spheroids and organoids provide physiologically relevant platforms for investigating multicellular interactions and tissue-level neural functions. In the following chapter, we discuss representative surface-engineered biosensors that extend biomarker monitoring beyond the cellular level to physiologically relevant spheroid and organoid models for disease modeling and therapeutic evaluation.

**Figure 4:**
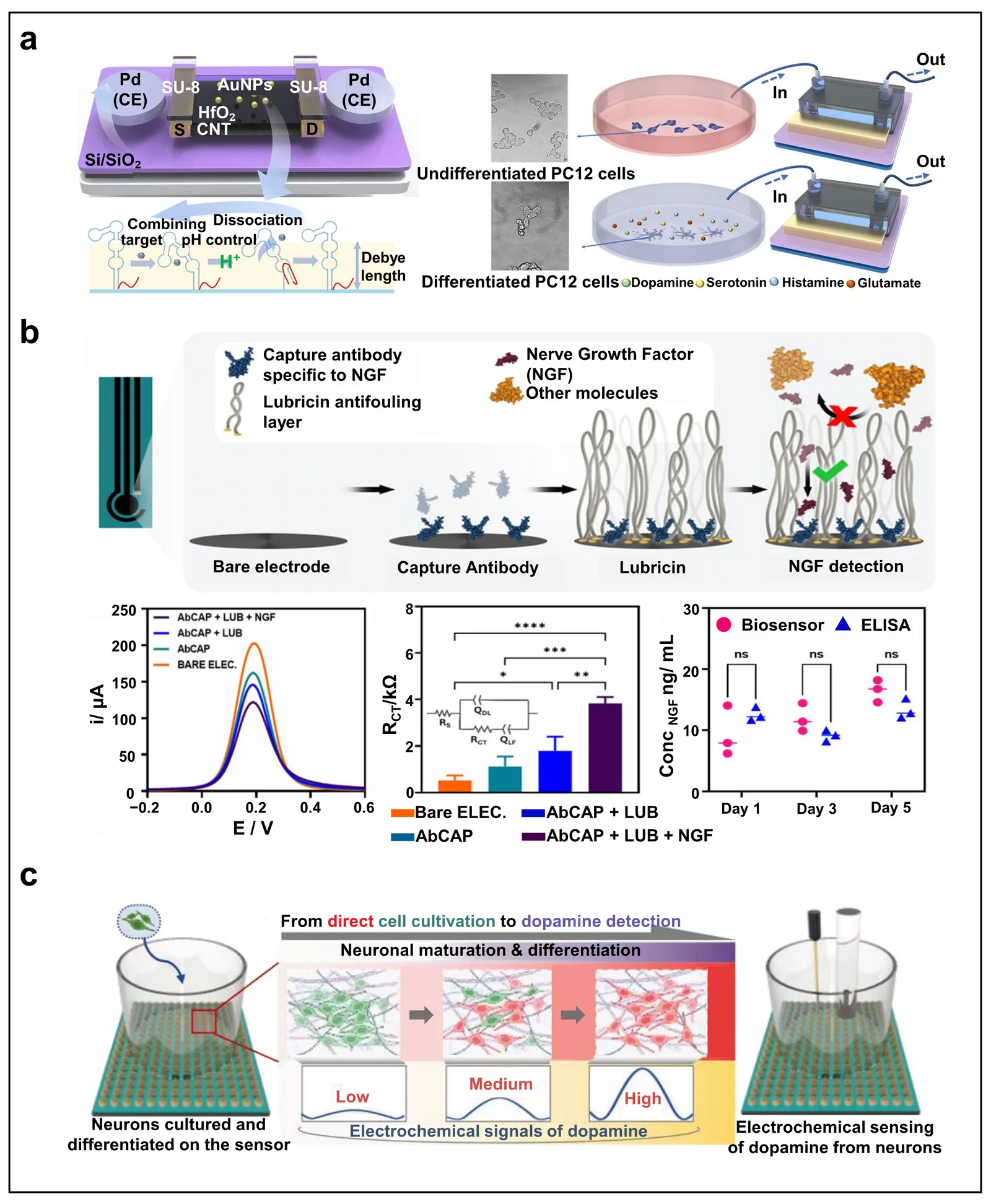
(**a**) Schematic illustration of an electrically resettable biosensor showing the reversible regeneration of the aptamer-functionalized sensing interface and multiplex detection of neurotransmitters released from living PC12 cells. Reproduced with permission from Ref. [132]. Copyright © 2025 The Author(s). Advanced Science published by Wiley-VCH GmbH. (**b**) Schematic illustration and electrochemical characterization of the PRG4-based antifouling immunosensor for NGF detection in cell culture media. Statistical significance between the groups was determined by one-way ANOVA (* *p* < 0.05, ** *p* < 0.01, *** *p* < 0.001, **** *p* < 0.0001; ns, non-significance). Reproduced with permission from Ref. [134]. Copyright © 2025 The Authors. Published by Elsevier B.V. (**c**) Schematic illustration of a GO-incorporated PPy/Au nanocluster hybrid nanopattern array for in situ electrochemical sensing of dopamine release from living neurons and functional assessment of stem cell-derived dopaminergic neurons. Reproduced with permission from Ref. [141]. Copyright © 2023 Wiley-VCH GmbH.

## 5. Surface-Engineered Biosensors to Detect Neural Biomarkers Beyond the Cellular Level for Biomedical Applications

Beyond the cellular level, spheroids and organoids have emerged as physiologically relevant in vitro models that more accurately replicate the structural organization, cellular heterogeneity, and functional connectivity of human organs than conventional 2D cell cultures [142]. Unlike conventional 2D cultures, these 3D systems generate complex biomarkers, such as neurotransmitters, extracellular vesicles, proteins, metabolites, and spontaneous electrical activities, for investigating neural development and neurological disorders [143]. However, the inherent structural complexity and delicate architecture of 3D tissues present unique challenges for stable interfacing with conventional biosensors, limiting continuous and non-destructive biomarker analysis. Consequently, surface engineering has emerged as a key strategy to bridge this gap. Surface engineering using bioadhesive polymers, SAMs, DNA nanostructures, and molecular recognition layers improve tissue immobilization and biomarker capture [144]. These advances have enabled surface-engineered biosensors to perform continuous and non-invasive monitoring of biomarkers beyond the cellular level for studying maturation, disease progression, and drug responses in physiologically relevant 3D models.

In particular, a surface engineering strategy has recently been studied to selectively detect pathogenic protein biomarkers secreted from neural organoid models. For example, an electrochemical biosensor based on peptide-imprinted poly(hydroxymethyl-3,4-ethylenedioxythiophene) (PEDOT-OH) nanotubes was developed for monitoring α-syn, a key pathological biomarker of PD [145]. The sensing interface was fabricated by electropolymerizing EDOT-OH in the presence of an α-syn-derived peptide template, followed by template removal to generate molecularly imprinted recognition cavities with high target specificity. The nanotubular conductive polymer significantly increased the effective electroactive surface area while facilitating rapid electron transfer for ultrasensitive electrochemical detection over a peptide concentration range of 0.065 pM–65 nM, while the full-protein assay showed an LOD and limit of quantification of 6.5 and 21.7 fM, respectively. The platform was subsequently applied to quantify secreted α-syn in the culture medium of patient-derived midbrain organoids, successfully distinguishing PD organoids from healthy controls. At day 38 of differentiation, the mutant organoids exhibited substantially higher extracellular α-syn levels (325.0 ± 8.5 fM) than healthy organoids (172.9 ± 8.0 fM), demonstrating the utility of molecularly engineered sensing interfaces for non-invasive monitoring of disease-associated protein secretion in physiologically relevant neural organoid models. The culture medium was diluted 1000-fold before sensing, and spike recoveries of 91.2 ± 4.7% to 96.7 ± 4.6% and retention of more than 94% of the initial response after six reuse cycles further supported measurement reliability. In addition to this study, surface engineering strategies have been extended to electrophysiological monitoring of complex brain assembloid models for cancer drug evaluation. A 3D electrophysiological biosensor was developed by integrating a glioblastoma (GBM)-midbrain assembloid with dual-channel flexible Au nanodot-patterned electrodes [146]. To enhance neural integration between the two organoids, the assembloid was assembled using an adhesive hydrogel incorporating neurotrophin-3 (NT3)-functionalized AuNP. The conductive NT3/AuNP hydrogel generated a bioactive interface that significantly enhanced electrical communication between the fused organoids, exhibiting 1.78 times higher electrical conductivity and 1.93 times lower resistance impedance than the bare hydrogel. These improvements promoted neuronal connectivity and resulted in an approximately two-fold increase in spontaneous electrophysiological spike activity. Furthermore, the flexible Au nanodot-patterned electrode increased the electrode–organoid contact area and enhanced the electrochemical response, enabling more sensitive electrophysiological recordings from individual organoids within the assembloid. The patterned electrode produced a redox current of 430 ± 0.69 μA compared with 35.5 ± 0.055 μA for the bare electrode. Importantly, the dual-channel design enabled simultaneous but independent sensing of the GBM and midbrain regions, allowing compartment-specific responses to be distinguished rather than averaged across the entire assembloid. Using this surface-engineered platform, the effect of Everolimus, an mTOR inhibitor for GBM treatment, was successfully evaluated. After one week of drug treatment, the electrophysiological signals of the GBM organoid decreased by approximately 2.46 times, demonstrating that the platform enables continuous monitoring of treatment-induced functional recovery and provides a promising strategy for organoid-based drug screening.

In addition to tumor spheroids and organoids, recent studies have increasingly focused on monitoring neurotransmitters released from brain organoids. Surface-engineered biosensors capable of continuous quantification of neurochemical signals provide valuable insights into neuronal maturation, synaptic function, and the progression of neurodegenerative disease. For example, a redox-cycling sensor with a surface engineering strategy using a SAM of 3-mercaptopropionic acid (3-MPA) was developed to detect dopamine secreted from the human midbrain organoids (hMOs) (Figure 5a) [147]. The thiol group anchored 3-MPA to the Au microsensor, whereas its negatively charged terminal group reduced albumin adsorption and interference from AA rather than directly capturing dopamine. The developed redox-cycling sensor was further applied to quantify dopamine released from hMOs. The modification increased Rct from 0.9 ± 0.1 to 3.5 ± 0.3 kΩ and improved dopamine selectivity over AA by 702%. For each analysis, 20 μL of pooled supernatant from three organoids was measured in technical triplicate using matrix-matched calibration. Intra- and inter-batch CVs were below 10%, sensitivity was stable for 12 days, and 20-month storage caused a 33% loss of sensitivity while retaining comparable selectivity. To confirm disease-associated changes in dopamine secretion, the study used healthy hMOs and genetically modified hMOs carrying a PD-related LRRK2 mutation generated using CRISPR/Cas9. Electrochemical signals provided a decrease in dopamine levels in LRRK2 mutant MO, reflecting a decline in the function of dopamine neurons. The organoids were then treated with 0.5 μM LRRK2 inhibitor II to evaluate the recovery of dopamine signals through a redox-cycling sensor. Although the treatment indicated no distinct change in healthy hMOs, the dopamine signal in the LRRK2-mutant organoids increased by approximately 87%, indicating a substantial recovery of the function of dopamine neurons. In addition, an electrochemical biosensor based on a mesoporous Au nanodot-patterned 3D concave electrode with a SAM of 3-MPA was recently developed for the sensitive detection of dopamine released from hMOs [148]. The mesoporous Au nano-dot-pattern indicated a capacitive current of 0.421 ± 0.016 mA, which was approximately 8.08 times higher than that of the bare electrode. This enhanced electrochemical signal enabled more sensitive and reliable quantification of dopamine levels over a broad concentration range from 1 μM to 10 mM. At 100 μM dopamine, the mesoporous electrode generated 167 ± 2.15 μC compared with 119 ± 6.07 μC for bare Au. Furthermore, the biosensor was applied to patient-derived iPSC-based MOs carrying PRKN or LRRK2 mutations to monitor drug-induced changes in the electrochemical signal of dopamine. After treatment with L-DOPA, a commonly prescribed medication for PD, the electrochemical signal of dopamine increased from 76 ± 1.9 to 150 ± 2.2 μC in PRKN-mutant organoids and from 71 ± 1.9 to 149 ± 2.6 μC in LRRK2-mutant organoids. These increases in signal demonstrate that the surface-engineered electrode can sensitively detect the recovery of dopamine secretion in disease-specific MOs. Beyond PD, surface-engineered biosensors have also been applied to the detection of AD-related biomarkers. In particular, tau protein, one of the key pathological biomarkers of AD, has been monitored using a SERS-based platform integrated with human cerebral organoids (hCOs) to investigate the apolipoprotein E (APOE)–tau axis (Figure 5b) [149]. For this, the Au-coated ZnO nanorod substrate was functionalized with half-antibodies through S-Au interactions, and antibody-conjugated AuNP nanotags further enhanced plasmonic hot spots. To achieve selective detection of tau protein, the SERS substrate was surface-engineered by immobilizing a monoclonal tau antibody onto a plasmonic Au nanostructure through strong S-Au interactions. This surface functionalization enabled stable antibody immobilization while preserving the plasmonic properties of the substrate. The platform distinguished total, aggregated, and phosphorylated tau using Raman bands at 300, 500, and 1050 cm-1, respectively. Such multiplexed sensing provides more comprehensive information on tau abundance, aggregation, and phosphorylation than a single-marker measurement. The total-tau assay achieved an LOD of 7.4 fM, and recoveries from spiked organoid culture medium ranged from 87% to 109%, with relative standard deviations below 4.5%. The antibody-functionalized SERS platform was subsequently employed to monitor tau protein secreted from hCOs, enabling non-invasive evaluation of AD-related pathological changes. The conditioned medium was collected at four-day intervals, enabling non-destructive longitudinal sensing without exposing the organoids to laser irradiation. By analyzing the SERS spectra of secreted tau, the platform successfully verified different developmental stages of hCOs. Furthermore, longitudinal monitoring revealed that tau secretion increased with organoid maturation and neurodegenerative progression before declining during astrocytic differentiation.

These studies demonstrate that surface-engineered biosensors have expanded neural biomarker detection beyond conventional cellular systems to more physiologically relevant spheroid and organoid models. By enabling sensitive and selective monitoring of secreted biomarkers from complex 3D neural tissues, these platforms provide valuable tools for disease modeling and therapeutic evaluation.

## 6. Conclusions and Future Perspectives

As neurological disorders emerge as a major global health challenge, it is essential to develop biosensing platforms that can detect important neural biomarkers in a sensitive and selective manner to be applied for the diagnosis of neurological disorders. In addition, if neural biomarkers are detected in an environment that mimics actual living organisms, this capability can be applied to evaluate biomarker levels in actual patients and individuals suspected of having neurological disorders in the near future. To achieve these goals, surface engineering strategies hold huge potential and applicability, providing new directions for developing accurate neural biosensors and further detect biomarkers at 3D cellular complex levels through the improvement of interfacial properties, facilitation of molecular recognition, suppression of nonspecific adsorption, and so on. Accordingly, to date, many surface-engineered biosensors have been developed by utilizing surface engineering strategies to detect neural biomarkers, including dopamine and Aβ, demonstrating their applicability for the diagnosis of neurological disorders.

In this review, we discussed recently reported research on neural biosensors utilizing surface engineering strategies to achieve accurate detection of neural biomarkers under various conditions. Table 2 presents a summary of the recent studies discussed comprehensively in this review. Based on the discussed research, a combination of nanostructured interface engineering and molecular recognition engineering is a suitable strategy for developing neural biosensors for single neural biomarker detection in a sensitive manner, conductive bio-interface engineering is suitable for biomarker sensing beyond the cellular level such as in organoids, and antifouling and passivation engineering is generally required for the fabrication of functional electrodes of biosensors. As reviewed here, even though different surface-engineered biosensors have been reported that are applicable in the diagnosis of neurological disorders, several issues should be further addressed for practical use. Most of the research discussed here is focused on analytical validation instead of clinical validation. For the diagnosis of neurological disorders of patients or suspected patients, clinical validation is required, and this entails resolving issues such as inter-patient biological variability, preanalytical sample handling, and longitudinal stability, which generally do not need to be considered during analytical validation. To achieve this goal, future research should move beyond in vitro analytical validation to enable the direct detection of biomarkers in actual patient blood; this entails achieving high detection accuracy [e.g., over 0.95 area under the curve–receiver operating characteristic (AUC-ROC) for Aβ] within a short evaluation time (e.g., within one hour). In addition, challenges include ensuring stability across diverse physiological environments, such as varying pH levels and surrounding ionic compositions, and achieving precise measurements without internal signal noise [150,151]. Particularly, from the perspective of practical use and clinical validation, the reproducibility issue, which is often overlooked at the educational or research laboratory level, should be addressed. For example, mass-production of advanced nanomaterials with homogeneity and reproducibility should be accomplished to develop neural biosensors in practical use, also considering the economic aspect. Furthermore, moving beyond single-target biosensing capabilities to enable multiplexed detection of various neural biomarkers holds the promise of developing more accurate neural sensors by simultaneously addressing the diversity of complex brain pathologies and the low accuracy of single biomarker detection [152]; however, the precise separation of measured multiple signals such as resolving signal overlaps is a critical issue that must be addressed. Also, the selection of suitable biosensing probes capable of selective detection among multiple targets is required for precise multiplexed detection. In addition, with the introduction of AI technology, which has recently received a lot of attention, more advanced AI-powered neural biosensors can be developed in the near future to address the measured signal overlapping issues that may arise during multiplex biomarker detection, including the precise simultaneous detection and quantification of multiple signals [153,154,155]. In conclusion, this review presents interdisciplinary knowledge and valuable insights for the development of newly advanced surface-engineered neural biosensors to be applied for the accurate diagnosis of neurological disorders in the early stage.

## Figures and Tables

**Figure 1:**
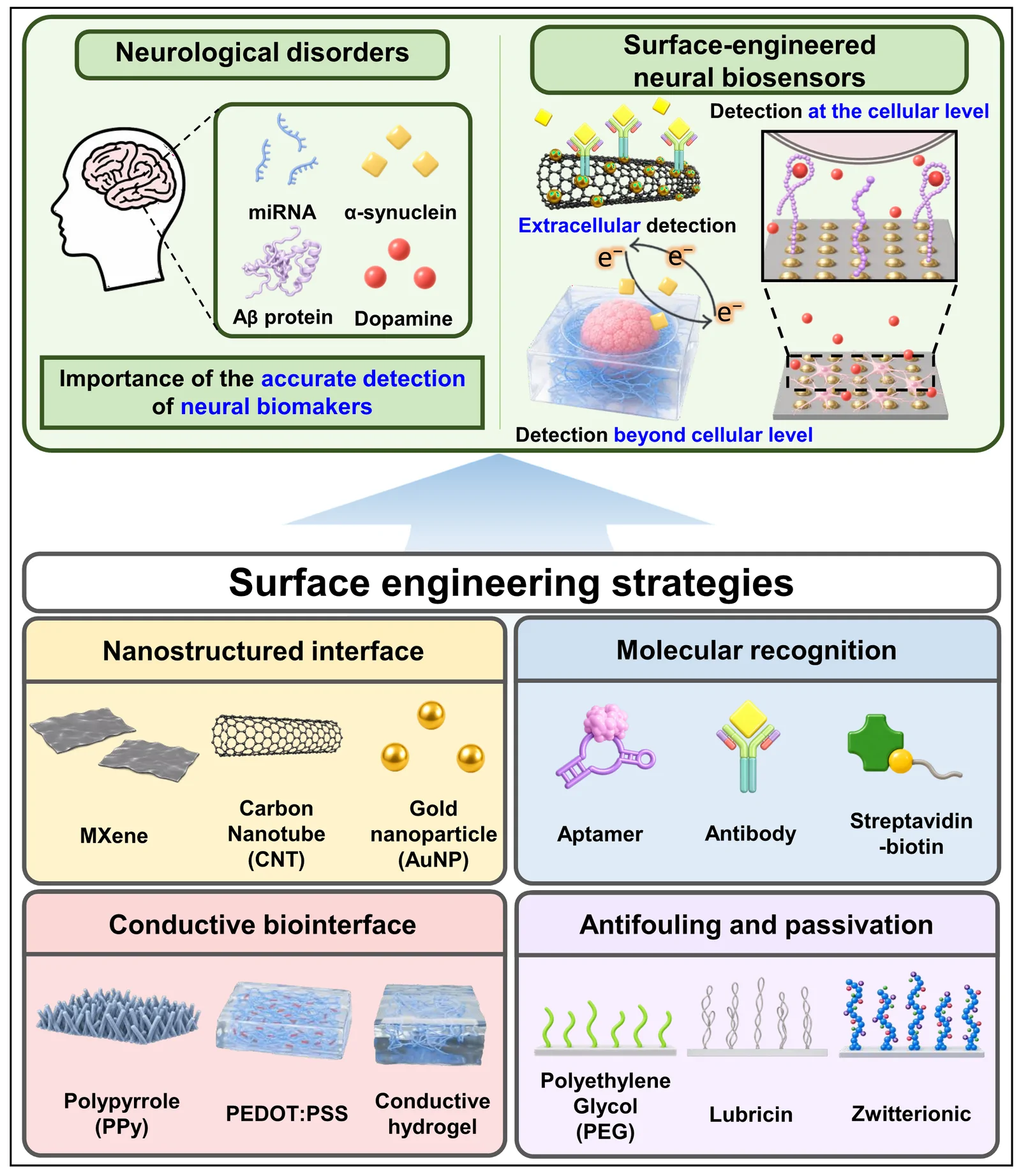
Overall schematic illustration of neural biosensors for the detection of biomarkers related to neurological disorders utilizing surface engineering strategies.

**Figure 5:**
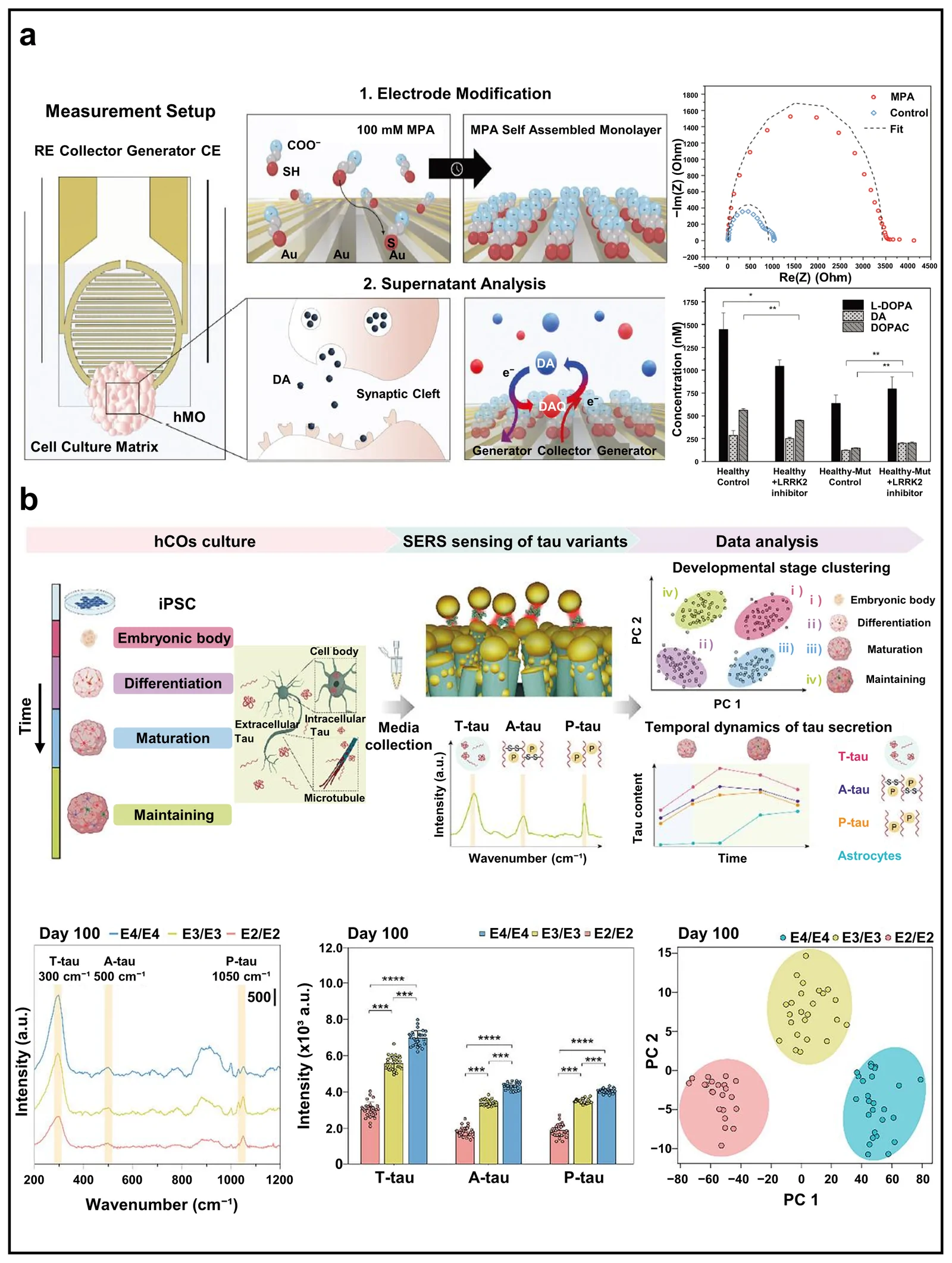
(**a**) Schematic illustration of an MPA-functionalized redox-cycling electrochemical microsensor for dopamine monitoring in hMOs and its application to disease modeling and drug screening. Statistical significance between the groups was determined by one-tailed *t*-test (* *p* < 0.05, ** *p* < 0.01). Reproduced with permission from Ref. [147]. Copyright © 2021 The Author(s). Published by the Royal Society of Chemistry (London, UK). (**b**) Schematic illustration of a surface-engineered immuno-SERS biosensor for monitoring AD biomarkers in hCOs. The platform enables quantitative analysis of tau protein variants and characterization of APOE genotype-dependent biomarker dynamics. Statistical significance between the groups was determined by Kruskal-Wallis test and Dunn's test (*** *p* < 0.001, **** *p* < 0.0001). Reproduced with permission from Ref. [149]. Copyright © 2025 The Author(s). Advanced Science published by Wiley-VCH GmbH.

**Table 2:** Summary of discussed studies on neural biosensors for the detection of biomarkers related to neurological disorders.

Sensing Environment	Surface Engineering Strategy	Probe	Target	Advantage	Performance	Ref.
Extracellularly	Molecular recognition engineering	Antibody immobilization on the electrode via streptavidin–biotin interaction	Aβ	Enhanced selective recognition of the target	Selectivity: 2.48 ± 0.19 nm (Aβ1-42) vs. 1.14 ± 0.11 nm (Aβ1-40) LOD: 12.0 fg/mL	[88]
	Molecular recognition engineering and Conductive biointerface engineering	PPhR-PPy composite-based nMIP	Tau protein	Signal amplification and enhanced target selectivity	Sensitivity: 8.99 μA/pg mL^−1^ vs. 4.39 μA/pg mL^−1^ (without PPy) Selectivity: 0.84 AUC vs. 0.81 AUC LOD: 498 fg/mL	[100]
	Molecular recognition engineering and Nanostructured interface engineering	Antibody immobilization on AuNP-electroplated electrode	α-syn	Enhanced electrochemical properties and sensitivity	Sensitivity: 1.78-fold (AuNP) LOD: 0.237 pg/mL	[103]
	Nanostructured interface engineering and Molecular recognition engineering	Zr/Ni-FcDA MOF and aptamer-modified glassy carbon electrode	Dopamine	Enhanced conductivity and target selectivity	Reproducibility: consistent across six sensors LOD: 0.07 μM	[110]
	Nanostructured interface engineering and Conductive biointerface engineering	Anti-miRNA immobilization on COF/Nafion-PEDOT:PSS modified electrode	miRNA	Enhanced conductivity and charge transfer	Recovery: 96–103% Long-term stability: 60 days LOD: 0.4 nM	[117]
At the cellular level	Nanostructured interface engineering	NC/MWCNT composite-modified electrode	Dopamine	Enhanced conductivity and dopamine chemisorption	Charge transfer resistance: kΩ → Ω range LOD: 1 nM	[129]
	Molecular recognition engineering and Nanostructured interface engineering	pH-sensitive aptamer on a semiconducting CNT film FET	Dopamine, serotonin, histamine, glutamate	Electrical resetting of the aptamer probe, enabling repeated real-time measurement	LOD: 10 fM Reset time: 1 min Reusability: stable responses over 10 cycles	[132]
	Antifouling and passivation engineering and Molecular recognition engineering	LUB-based antifouling layer with anti-NGF capture antibody on a SPE	NGF	Size-selective exclusion of large interfering proteins	LOD: 0.048 nM vs. 0.95 nM (without LUB) Sensitivity: 11.202 kΩ vs. 7.061 kΩ	[134]
	Antifouling and passivation engineering and Molecular recognition engineering	Cholesterol-anchored aptamer on brain cell membrane-coated chitosan-modified CFE	Dopamine	Antifouling with preserved aptamer-mediated selectivity	LOD: 5 nM Antifouling: 3% current decrease under BSA vs. 60% for bare CFE	[135]
	Conductive biointerface engineering and Nanostructured interface engineering	GO-integrated PPy/Au nanocluster 3D nanopillar array on ITO	Dopamine	Enhanced dopamine adsorption and signal amplification	Sensitivity: 2.35-fold (Au nanocluster) LOD: 33.4 nM Reproducibility: stable over six scans	[141]
Beyond cellular level	Nanostructured interface engineering and Molecular recognition engineering	Peptide-imprinted PEDOT-OH nanotube	α-syn	Enhanced target specificity and increased electroactive surface area	LOD: 6.5 fM Spike recovery: 91.2 ± 4.7% to 96.7 ± 4.6%	[145]
	Nanostructured interface engineering and Conductive biointerface engineering	NT3/AuNP hydrogel with flexible Au nanodot-patterned electrode	Electrophysiology	Enhanced electrical coupling between fused organoids	Conductivity: 1.78-fold increase (vs. bare hydrogel) Impedance: 1.93-fold decrease	[146]
	Antifouling and passivation engineering	3-MPA SAM on Au microsensor	Dopamine	Reduced albumin adsorption and AA interference	Charge transfer resistance: 0.9 ± 0.1 → 3.5 ± 0.3 kΩ Selectivity: 702% improvement over AA	[147]
	Nanostructured interface engineering and Antifouling and passivation engineering	Mesoporous Au nanodot-patterned 3D concave electrode with 3-MPA SAM	Dopamine	Increased surface area and electrochemical activity with antifouling	Capacitive current: 8.08-fold (vs. bare electrode) LOD: 1 μM	[148]
	Nanostructured interface engineering and Molecular recognition engineering	Au-coated ZnO nanorod conjugated with a half-antibody	Tau protein	Multiplexed detection via distinct Raman bands	LOD: 7.4 fM Recovery: 87–109% (RSD < 4.5%)	[149]

## Data Availability

No new data were created or analyzed in this study. Data sharing is not applicable to this article.
